# Increasing Parental Knowledge About Child Feeding: Evaluation of the Effect of Public Health Policy Communication Media in France

**DOI:** 10.3389/fpubh.2022.782620

**Published:** 2022-02-24

**Authors:** Sofia De Rosso, Pauline Ducrot, Claire Chabanet, Sophie Nicklaus, Camille Schwartz

**Affiliations:** ^1^Centre des Sciences du Goût et de l'Alimentation, CNRS, INRAE, Institut Agro, Université de Bourgogne Franche-Comté, Dijon, France; ^2^Santé publique France, French National Public Health Agency, Saint-Maurice, France

**Keywords:** public health nutrition, child feeding, knowledge assessment, feeding guidelines, nutrition education

## Abstract

**Background:**

Unhealthy eating behaviors are risk factors for non-communicable diseases. Parents largely influence the development of eating behaviors during childhood through their feeding practices. Parental feeding practices in line with recommendations are more likely to turn into healthier outcomes in children. From a public health perspective, it should be first ascertained whether providing parents with recommendations about child feeding is a useful approach for increase parental knowledge. Recently, the French health authorities developed a brochure covering updated child feeding recommendations. The present study aims to evaluate the short-term effects of reading this brochure on parental knowledge about child feeding, distinguishing knowledge accuracy and certainty.

**Methods:**

A brochure containing updated child feeding recommendations for 0–3 years old was developed by the French public health agency. A representative sample of French parents (*n* = 400) was targeted to complete an online questionnaire (T0) comprising 30 statements regarding child feeding. For each statement, parents indicated whether it was true/false and how certain they were of their answer (4-point scale). After receiving and reading the brochure, the same parents completed the same questionnaire 3 weeks later (T1). Accuracy (number of correct answers) and certainty (number of mastered answers: correct answers given with the maximal degree of certainty) were compared at T1 vs. T0 using paired *t*-tests. Knowledge evolution based on parental age, parity and education level was tested with linear models.

**Results:**

A total of 452 parents responded at T0 and T1 and were considered for analysis. Between T0 and T1, the number of correct answers [median 22–25, *t*_(451)_ = 17.2, *p* ≤ 0.001] and mastered answers [median 11–17, *t*_(451)_ = 18.8, *p* ≤ 0.001] significantly increased. The median of the difference between T1 and T0 was larger for mastered than for correct answers. The observed evolution in knowledge was independent of parental age, parity or education level.

**Conclusions:**

A brochure containing child feeding recommendations has the potential to increase the accuracy and, to an even greater degree, the certainty of parental knowledge. This increase was observed even for younger or less educated parents.

## Introduction

Childhood obesity is a major public health concern worldwide, threatening the health of children, especially in Western societies. In France, in 2013, 12% of children under the age of six were overweight (1); moreover, in 2017, 18% of adolescents were overweight, and 5% were affected by obesity. More of concern, between 2009 and 2017, the prevalence of childhood obesity rose (2), despite the deployment of public health campaigns starting in 2001 to target nutrition and physical activity (3). The benefits of establishing healthy eating behaviors from early childhood are countless in terms of non-communicable disease prevention. In fact, previous studies have demonstrated that health-promoting nutritional practices in the first 1,000 days of life can positively impact future health (4). For example, the introduction of vegetables can be boosted during the early years of a child through the application of specific feeding practices, fostering their acceptance later on (5). Furthermore, food preferences established early in life track into adulthood and are the basis for pursuing the maintenance of a healthy diet (6). Parental feeding practices influence the development of children's eating habits and preferences (7, 8). Those practices have an impact on shaping the child's risk of developing diet-related, non-communicable chronic diseases, such as obesity.

International and national feeding guidelines aim to facilitate the familiarization of parents with evidence-based best practices and guide them in the feeding process; however, two main problems might arise. First, from a public health perspective, it is difficult to always maintain the recommendations in line with the latest scientific evidence. Some countries may have incomplete feeding recommendations due to a lack of regular updating (9). Consequently, public health stakeholders may face challenges in terms of institutional time required for national nutrition and health policies renovation. For instance, in France, the communication material used to spread the official child feeding recommendations is not recent (2004), despite new guidelines covering feeding children 0–3 years of age having recently been published, but not yet adapted toward lay public dissemination (10). Second, having updated guidelines does not automatically leads to knowledge increase and changes in behavior. Care should be given in bridging the existing gap between the evolution of scientific knowledge and the transfer of this evidence-based knowledge in a timely manner into public health policies and actions (e.g., communication campaigns, interventions on the environment). Ultimately, this should be beneficial for promoting healthy behaviors changes within the population.

Feeding practices of French parents do not always meet official recommendations. A cross-sectional study conducted in 2013 with a sample of 1,184 children under the age of 3 years increased focus on the low prevalence and duration of breastfeeding (11). Regarding the introduction of solid foods, the same study showed that 54% of infants were introduced to solid foods between 4 and 6 months of age, but younger mothers still struggled with achieving the initiation of complementary feeding within the recommended time frame (11). Low breastfeeding duration was observed in another French cohort and was shown to be linked to a variety of factors. Those factors included the fact that mothers had been breastfed themselves, and a high rate of maternal professional activities (12–14). Data from the French study Epifane (a nationwide birth cohort) confirmed that socioeconomic characteristics can affect the concordance of complementary feeding behavior with national recommendations. In fact, parents in more disadvantaged situations were more likely to follow less strictly the guidelines (15). Similar results emerged from studies conducted in other European countries, with the introduction of solid foods occurring earlier than recommended by national guidelines (16). Parental cultural and sociodemographic characteristics (e.g., lower socioeconomic status and education level), or other markers of unhealthy lifestyle (e.g., maternal smoking), often predicted the early start of complementary feeding (16–18).

A recent integrative review showed that European parents' knowledge about child feeding, particularly complementary feeding (e.g., when to first start the introduction of solid foods), is far from optimal (19). In contrast, an online quantitative survey conducted in the UK evaluated parents' understanding of feeding guidelines, reporting high knowledge of the recommendations (20). Providing information is one means to increase knowledge, but increasing knowledge is not sufficient to predict a change toward healthier behaviors (21). Nevertheless, knowledge remains one of the main components of the theoretical domains framework to achieve behavior change when implementing health interventions (22). Therefore, understanding information and comprehending recommendations remain important steps in the early-stage process of eliciting lifestyle changes through behavior modifications. One major challenge is to define the best strategy to educate parents toward feeding practices that encourage the adoption of healthy eating behaviors in children. Many different theories have been applied to explain parent feeding practices, including ecological systems theory (23) and social cognitive theory (24). Nevertheless, little is known about the knowledge that might drive parents to adopt a certain practice. Metacognition theories explore how the “feeling of knowing” can mediate controlled vs. automatic processes, implying that a difference might exist in applying knowledge that we might be more or less conscious to have acquired (25). A study performed by Bruttomesso et al. found that, in order to better characterize knowledge, subjects can be asked to indicate their degree of certainty for each answer (26). A similar approach has been used by Norman and Furnes to measure confidence rate when answering questions (27). To the best of our knowledge, no similar research has been done in the context of understanding parental perception of child feeding guidelines, highlighting a gap in the literature. It is hypothesized that being certain of one's own (correct) knowledge can make one more inclined to apply it (26). In this context, when evaluating knowledge, it seems important to understand both the correctness (accuracy) and the degree of certainty.

In France, public health stakeholders have been preparing a new communication strategy regarding infant and young child feeding before its field implementation. A first step was the renewing the official child feeding guidelines (10, 28). Then two quantitative studies were performed to understand French parents' and pediatricians expectations on communicating about child feeding information (29, 30). Among the support measures employable for public health dissemination purposes, paper documents have been shown to continue to play a role: 44% of parents expressed using them when seeking child feeding information (30). For 59% of pediatricians, paper brochures appeared to be the most effective tool for grabbing parents' attention when advising them about feeding during consultations (29). Santé publique France (the public health agency of the French Ministry of Health) then developed a paper brochure containing the latest child feeding recommendations, as an attempt to spread new child feeding recommendations efficiently to parents. Parents qualitatively tested this brochure (focus groups and individual interviews). In this context, it was judged essential to characterize whether this brochure could make it possible to increase parental knowledge accuracy and degree of certainty. In addition to evaluating the global effect of reading the brochure, this approach should also make it possible to identify which, if any, of the specific topics the parents were more uncertain about. Within this framework, considering the existing gap in child feeding knowledge evaluation, the present study aimed to evaluate: (1) how much French parents know about child feeding (accuracy and certainty of knowledge); (2) whether the brochure containing new official child feeding recommendations could contribute to increasing parental knowledge (accuracy and certainty) about child feeding; and (3) whether knowledge evolution related to reading the brochure would depend on parental sociodemographic characteristics. A secondary objective was to explore parental attitudes toward the content of the brochure.

## Materials and Methods

### The Updating of Child Feeding Recommendations and the Development of a Brochure Targeted to Parents

In France, official recommendations for feeding children ages 0–3 years have been updated and published recently by ANSES (the French national agency for Food, Environmental and Occupational Health Safety) under the supervision of the Ministry of Health (10). In October 2020, the High Council of Public Health released a report reflecting these benchmarks (28). As of follow-up in 2019–2020, the content for a paper brochure intended for the final users of this communication strategy, the parents, had been developed by the French public health agency, Santé publique France. The intent was to make the recommendations relating to feeding children ages 0–3 years as accessible as possible to parents. In the present study, the document that was sent to the parents was a draft version of the final brochure, and it comprised just the core text, without subsequent graphical adaptations. The brochure contained 11 pages plus a table that summarized the recommendations for introduction of each food group based on the age of the child. The brochure was divided into ten chapters. Five chapters addressed the topic of feeding based on the age of the child (milk feeding, complementary feeding until 3 years of age when the child eats like the whole family); three chapters covered specific topics in line with parental feeding practices (e.g., responsive feeding); one chapter addressed physical activity and sleeping; and one chapter summarized foods not suitable for children. The version of the brochure (in French) used for this study is presented as Supplementary Material.

### Participants, Questionnaire, and Study Procedure

**Figure 1:**
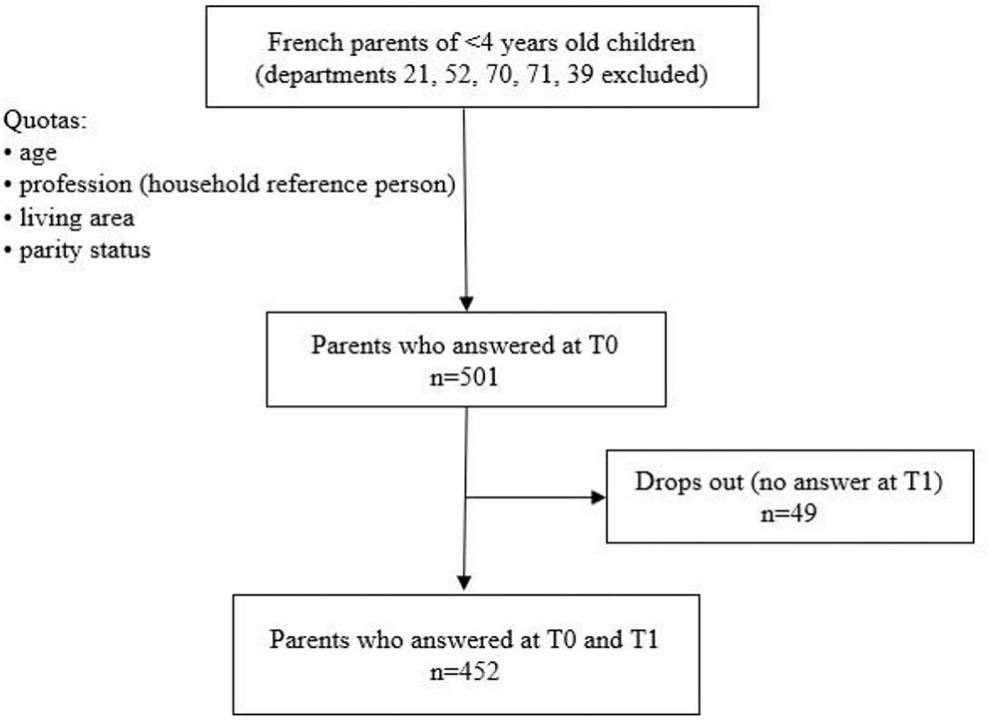
Flowchart of the participants.

The recruitment was done via an agency (Panelabs – MIS Group) composed of a panel of participants from all around France. We could not run a power calculation to set the population size in the absence of a previous study on this topic, but a targeted sample size of 400 was defined *a priori*, as it was considered large enough for our purposes based on previous studies conducted by Panelabs on similar subpopulations. The recruiting agency aimed to initially include a sample of 500 parents to account for potential drops out (see Figure 1 for the flowchart of participants). The targeted population comprised French parents of children between 0 and 3 years of age. Specific prerequisites to participate were: (1) having a child < 4 years old and (2) not residing in one of the following French departments: 21, 52, 70, 71 or 39 (to avoid biases due to the implementation of another study in these areas). The representativeness of the sample was ensured by the quota sampling method, which was applied to the study population (parents of 0–3 years old children living in France) on the following variables: age of the parent; profession of the household reference person (i.e., the parent with the highest salary); living area (urban vs. rural); and primiparous or multiparous parent. The general population census was used to identify the quotas within our study population and for data calibration (31).

To collect the data, two online questionnaires were prepared by the researchers and were administered online to the participants via the web system of the recruitment agency (Made in Surveys) at two different time points. The first online questionnaire (T0) comprised an initial part to collect demographic data and a second part made up of 30 items in the form of declarative sentences to evaluate the child feeding knowledge of parents (see detailed content below and Table 1 for the items). The second questionnaire (T1) was made up of the same 30 items plus some questions to gather other information regarding the attitudes of parents toward the brochure (i.e., usefulness of the content and parental self-efficacy regarding following the recommendations of the brochure). The questionnaire was checked for accuracy and appropriateness by two experts working at Santé publique France in October 2020, to ensure that all the topics of the brochure were covered by the questionnaire. At the same time, it was also certified that all the novel aspects of the recommendations were covered by the items; to check for this, the items were classified as new, old or reformulated notions (in comparison to the previous guidelines) and checked by two of the authors. The questionnaires were pretested by the recruitment agency with 41 participants. After the pretest the questionnaire was sent to all the other participants. Quality of the answers was ensured by the fact that all of the questions required a mandatory answer, therefore there was no risk for uncompleted questionnaires.

**Table 1:** Items exploring parental knowledge about child feeding.

**Q**	**Q code**	Item^*^	**True**	**False**
1	Only milk < 4 m	Until the age of 4 months, a baby should be given only milk, nothing else.	X	
2	Growup milk 6–12 m	Growing-up milk is suitable for babies aged 6 to 12 months.		X
3	Almond milk	Almond milk is suitable for the baby's needs, as long as it is fortified with calcium.		X
4	Milk alternance	From 1 year, it is possible to give alternately “growing-up milk” and whole UHT cow's milk.	X	
5	All foods 4–6 m	All foods can be given between the 4 and 6 months of the child [vegetables, meat, fish, fruits, eggs, pulses (lentils, beans, chickpeas), starches including whole starches (pasta, rice, semolina, bread), dairy products, unsalted nuts (almonds, hazelnuts, walnuts)] in the order you want but adapting the texture to the child's age.	X	
6	New textures 6–8 m	Between 6 and 8 months, most babies are able to swallow smooth purees without any problem and are ready to eat new textures.	X	
7	Drinks	At the start of complementary feeding, it is advisable to start giving the child different types of drinks such as fruit juice or plant-based milks (i.e., almond milk).		X
8	Food refusal	If a child does not like a food after 2 or 3 tries, there is no point in continuing to offer him that food.		X
9	Reward	It is advisable to offer small rewards (toys, desserts, etc.) to encourage the child to finish all the vegetables on his plate.		X
10	Veg variety	It is important to give the child a taste of a wide variety of vegetables, varying the recipes.	X	
11	Veg diet only	It is possible to offer a vegetarian or vegan diet to children under 3 years old.		X
12	Finish food	If a child does not finish what he has on his plate, it is good to force him to finish because he needs to eat everything to be healthy.		X
13	Screen < 3 y	Exposing a child under the age of 3 to any screen (TV, tablet, smartphone) is not recommended.	X	
14	Moving	It is advisable to encourage the baby to move, especially with games, from 6 months of age.	X	
15	Force	If a child refuses a food, he should not be forced to eat it.	X	
16	Growth chart	The best way to tell if a child is eating well and getting enough is to follow the growth chart during visits to the doctor.	X	
17	Family food	When the child comes to the table with his family, he can eat just like everyone else.		X
18	Pulses 2/w	From the age of 1 it is advisable to offer the child pulses (lentils, chickpeas, beans) at least twice a week.	X	
19	Fats	Fats (such as a teaspoon of oil) should always be added to homemade preparations and store-bought foods if they do not contain it.	X	
20	Raw milk	Raw milk products and raw milk cheeses may be offered to children under 3 years old.		X
21	Juices	Fruit juices are one of the foods that must be introduced into the child's diet at the start of complementary feeding.		X
22	Water only	The only recommended drink (other than milk) for a child up to 3 years old is water.	X	
23	Salt	It is recommended to add salt to “homemade” foods.		X
24	Whole starch	It is possible to introduce whole starch foods (pasta, rice, semolina, bread) and pulses (lentils, beans, chickpeas) in the child's diet from the start of complementary feeding.	X	
25	Nut powder	Unsalted nut powder (almonds, walnuts, hazelnuts) can be added in a puree or a compote.	X	
26	Allergens	To avoid the risk of allergy, the main food allergens (such as eggs and peanuts) should not be introduced at the start of complementary feeding.		X
27	Tasks division	Parents decide what and when to eat while the child decides how much to eat.	X	
28	Neophobia 2 y	It is normal for children to begin to refuse new tastes or new textures around the age of 2.	X	
29	Wake up	If the baby falls asleep on the bottle, wake him up to finish all the contents of the bottle.		X
30	Bottle to sleep	Leaving a bottle in your baby's bed or leaving him in front of the TV are good strategies to let him falling asleep.		X

* *Items translated from French to English*.

*The correct answer is indicated for each item*.

*Beside answering True/False to each item, parents had also to indicate how sure they were about their answer, choosing between: absolutely not sure, rather not sure, rather sure, very sure*.

The T0 questionnaire was sent to the participants on November 13th, 2020. The exact date of completion was registered for each participant. Immediately after completion the participants received an email with a PDF version of the brochure, and the paper version of the brochure was also sent to each participant by regular mail. The last answers to T0 were obtained on November 24th, 2020, and by November 25th, all the paper brochures were sent to the participants. The T1 questionnaire was then sent by December 8th, 2020 to all the participants who answered at T0; in this way, it was estimated that all the parents had at least 2 weeks to read the brochure before completing the second questionnaire. The last answers to T1 were obtained on January 4th, 2021.

### Measures

#### Demographics

Parents were asked to report how many children they had and specify the sex and the date of birth of each child to ensure a precise calculation of each child's age. Parents were also asked to report their own sex, age, living area, employment status, number of persons in the household, number of years living in France, whether French was their mother tongue, level of education and perception of their financial status. In addition, to describe the feeding history of their youngest child, parents were asked to report whether the youngest child was born preterm, had medical problems that could have impacted his or her diet (medical conditions: gastroesophageal reflux disease, cow's milk protein allergy, nasogastric intubation or congenital abnormalities of the digestive tract), was breastfed (if yes, for how long), and had started complementary feeding (if yes, what was the frequency of given commercial and homemade baby foods).

#### Parents' Knowledge (Accuracy and Degree of Certainty)

The questionnaire items exploring parental knowledge were in line with the content of the brochure, covering the ten chapters. The 30 items were developed to ensure that all of the chapters of the brochure were covered, and special attention was given to those recommendations that differed from previous guidelines. In fact, 17 of the 30 items covered aspects that were not addressed in the previous recommendations (32) and were thus considered new. Following results from a previous survey, it was ensured that all the content that parents were looking for was also covered (30). The content can be categorized into one of the following six areas: (1) breastfeeding/milk feeding (four questions); (2) age and modalities of introduction of food groups and different textures (three questions); (3) feeding strategies (four questions); (4) child behavior and parental feeding practices (six questions); (5) recommended foods (nine questions); and (6) children's cues (four questions). The questionnaire (list of items) is shown in Table 1. For each item, participants were asked to indicate whether it was true or false and to score their degree of certainty on a four-point scale (absolutely not sure, rather not sure, rather sure, very sure).

#### Parental Attitudes Toward the Content of the Brochure

In the second part of the T1 questionnaire, parents were asked their opinion regarding the content of the brochure. They were asked to indicate whether they found the content to be useful, easy to understand, to answer their questions and whether the topics that they considered interesting were well covered in the brochure using a 4-point Likert scale, which ranged from strongly agree to strongly disagree. Parents were also asked whether, in the prior 2 weeks, they had sought information regarding child feeding anywhere other than in the brochure and, in the case of an affirmative answer, in which media they did so. Finally, parents were asked to answer using the same 4-point Likert scale whether they agreed on three self-efficacy statements regarding following the recommendations contained in the brochure. In particular, they were asked to indicate whether: (1) they were willing to follow those recommendations; (2) it would be difficult to follow the recommendations without the support of their partner and family; and (3) it would be difficult to follow the recommendations if their friends were not following the same recommendations. These three self-efficacy statements were formulated in accordance with a validated questionnaire to assess maternal attitudes toward infant feeding (33).

### Ethical Consideration

The study was conducted according to the guidelines laid down in the Declaration of Helsinki. Participants voluntarily agreed to participate in the study, gave their informed consent to take part in the study (by ticking a box on the first page of the questionnaire), and were compensated for their participation according to the criteria of the recruiting agency. The compensation was set at 3.80 euros for all finalized respondents (who went to the end of the process, with T1 completed at 100%). This amount was credited to the participant's account on the website of the recruiting agency. This study was approved by the institutional review board (IRB00003888, IORG0003254, FWA00005831) of the French Institute of Medical Research and Health, and a study registration was performed by the relevant data protection service.

### Statistical Analysis

For all statistical analyses, R version 3.6.1 was used (34). Frequencies, percentages and medians with interquartile ranges (IQRs) were used to describe the results. The statements regarding parental attitudes requiring an answer on a 4-point Likert scale were considered discrete and were dichotomized as “agree” (grouping the two positive answers) or “disagree” (grouping the two negative answers) for the analysis. An answer was considered correct when parents gave the right true/false value and mastered when parents gave the correct answer with a higher degree of certainty. A global score of knowledge was calculated by summing the number of correct answers of all the questions (range, 0–30). The same global score was also calculated for mastered answers. The difference in the number of correct answers between T0 and T1 (T1–T0) was considered to define the evolution of knowledge accuracy; knowledge certainty evolution was defined by the difference in the number of mastered answers between T0 and T1 (T1–T0). Paired *t*-tests were performed to determine whether the mean difference in correct and mastered answers between T0 and T1 was significant. McNemar's tests were calculated to check whether the proportion of correct answers (and mastered answers) differed significantly for each individual item between T0 and T1. The effect of selected sociodemographic characteristics (parent age, parity and education level) on knowledge (accuracy and certainty) at T0 and evolution between T0 and T1 was tested with a linear model. One model per variable was run to verify the effect of each characteristic on the number of correct and mastered answers at T0 and on the difference in correct and mastered answers between T0 and T1.

For each question, the proportion of correct answers was compared to 0.5, the chance level, through tests based on the normal approximation of the binomial distribution. Two kinds of tests were performed. First, a unilateral test was performed at T0 to detect recommendations opposite to the common belief. The alternative was a probability lower than 0.5, and rejection of the null hypothesis was an indication of disagreement with the general conviction. Second, another unilateral test was performed at T1 to detect questions that clearly ought to be reworked. The alternative was a probability higher than 0.5, and no rejection of the null hypothesis was an indication of response at chance level. When appropriate, the χ^2^ test was used to determine whether the relationship between parental attitudes toward the content of the brochure and sociodemographic characteristics was statistically significant. Significance was set at *p* < 0.05.

## Results

### Characteristics of the Study Sample

The characteristics of the study sample are described in Table 2. A total of 501 parents responded at T0, but only 452 parents also responded at T1 and were considered for the analysis (Figure 1).

**Table 2:** Characteristics of the sample of French parents who responded to the survey at T0 and T1 (*n* = 452).

**Characteristics**		***N***	**%**
**Responding parent's characteristics**			
Gender	Female	365	81
	Male	87	19
Age	<35 years old	253	56
	35 years old and more	199	44
Education level^a^	< A level	87	19
	≥A level	365	81
Socioprofessional category of the interviewed parent^b^	Low	211	47
	High	146	32
	No occupation/retired	95	21
Parity	Primiparous	152	34
	Multiparous	300	66
**Younger child characteristics**			
Prematurity	Yes	40	9
	No	412	91
Ever breastfed	Yes	295	65
	No	157	35
Complementary feeding started	Yes	413	91
	No	39	9
Having problems that could affect the diet	Yes	26	6
	No	426	94
**Household characteristics**			
Self-perception of financial situation^c^	Good	266	59
	Difficult	186	41
Living area	Rural	191	42
	Urban	261	58
Household composition (median = 4 people)	Single parents with children	29	10
	Couple with children	423	90

a *A level corresponds to the diploma obtained after completion of upper secondary school (equivalent to 12 years of formal education in France)*.

b *High (liberal profession, entrepreneur, executive or higher intellectual profession), intermediate or low (laborers and clerks) or no occupation/retired (including also students)*.

c *Parents were classified as having a good financial situation when they perceived they were comfortable or okay with it*.

*The other parents were classified as being in a difficult financial situation when they had a perceived uncomfortable situation imposing to pay attention to their budget, or making it difficult to reach the end of the month or forcing them to take out debts*.

### Evolution of Knowledge Accuracy and Degree of Certainty Between T0 and T1

**Figure 2:**
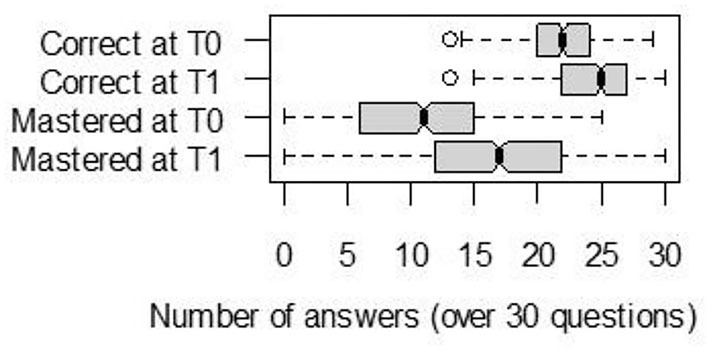
Median, lower and upper quartiles, of correct and mastered answers, over the 30 questions about complementary feeding answered by *n* = 452 parents at T0 and T1.

In our sample of parents, knowledge accuracy and knowledge certainty significantly improved after reading the brochure. In fact, the number of correct answers increased from 22 (IQR = 4) at T0 to 25 (IQR = 5) at T1, and the number of mastered (certain) answers increased from 11 (IQR = 9) to 17 (IQR = 10) (Figure 2). Paired t-tests showed that both differences were significant [correct answers: *t*_(451)_ = 17.15, *p* < 0.001; mastered answers: *t*_(451)_ = 18.81, *p* < 0.001]. At T1, the variability in the number of mastered answers (ranging from 0 to 30) was higher than the variability in the number of correct answers (13–30).

**Figure 3:**
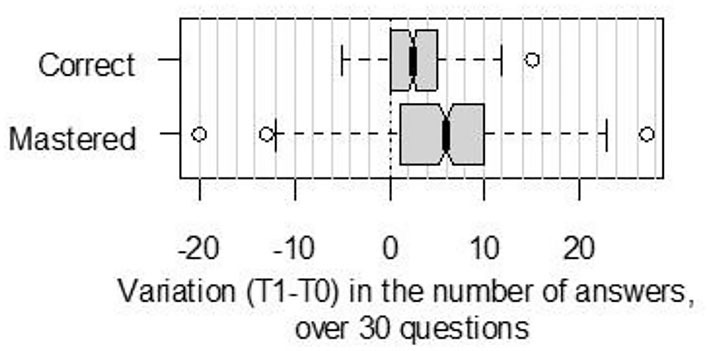
Variation in the number of correct and mastered answers, between T0 and T1 (before/after reading the brochure), for *n* = 452 French parents who rated the correctness of 30 questions (true/false), and rated their degree of confidence for each answer (mastered = correct answer given with the maximal degree of confidence, on a 4-pt scale).

Figure 3 shows the distribution of answer evolution between T0 and T1. For 75% of parents, the number of correct answers increased, and for more than 75% of parents, the number of mastered answers increased.

No significant effect of the parents' sociodemographic characteristics (age, education level and parity status) was found on the evolution of knowledge between T0 and T1. At T0, an effect of education on the proportion of mastered answers was observed. Parents with a higher education level had a significantly higher mean of mastered answers at T0 than did parents who had fewer years of formal education (mean difference = 1.4; *t*-value = 2.14; *p* = 0.03). The effects of other sociodemographic characteristics on knowledge accuracy or degree of certainty at T0 were not significant (all *p*-values > 0.05).

### Knowledge Accuracy and Certainty at T0 and T1 for Each Question

**Figure 4:**
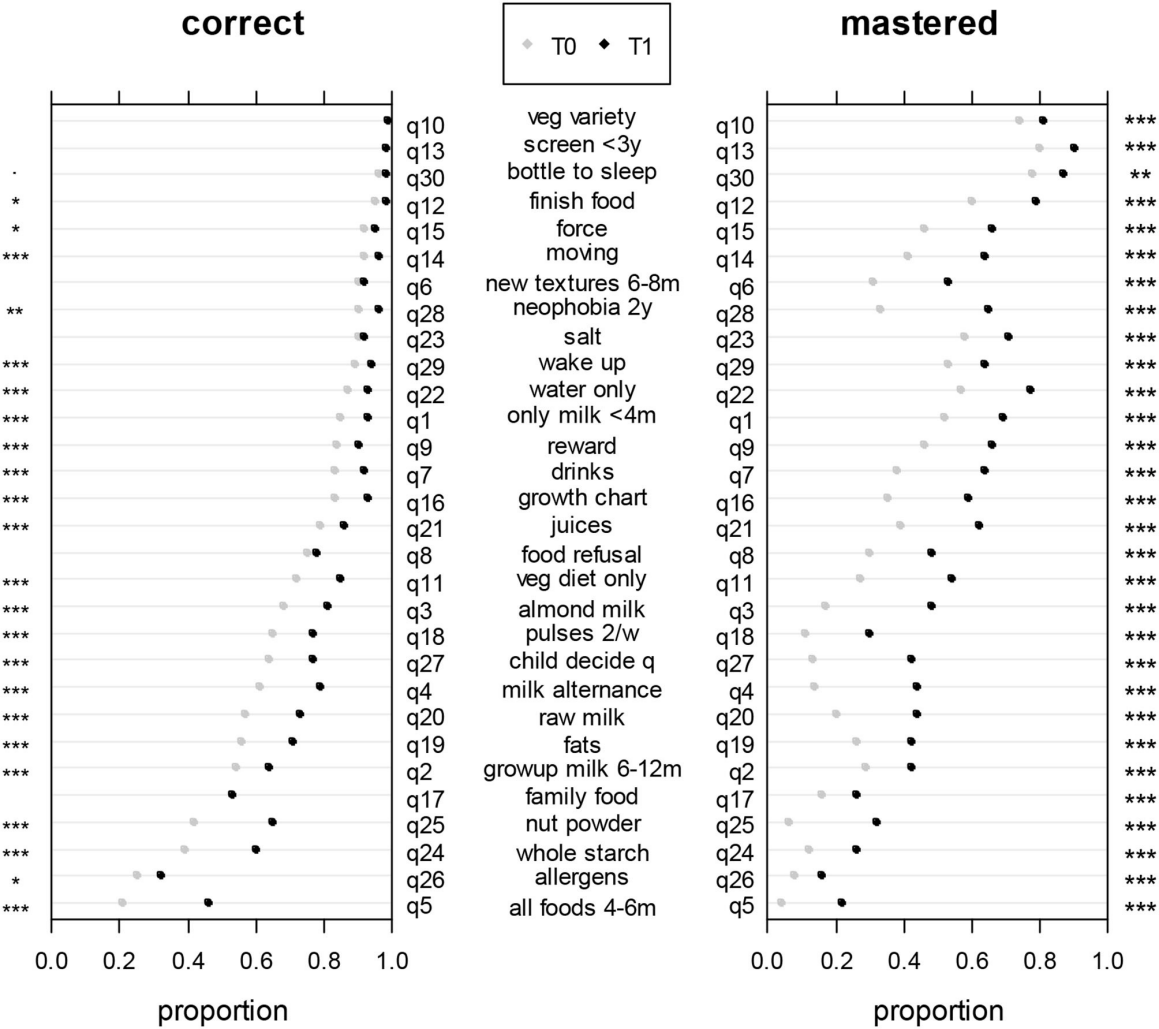
Proportions of correct answers (on the left) and mastered answers (on the right) at T0 (gray dot) and T1 (black dot) for each question. The asterisks indicate whether the increase between T0 and T1 was significant (McNemar's tests). *p < 0.05; **p < 0.01; ***p < 0.001.

Figure 4 shows the evolution of knowledge between T0 and T1 for each of the 30 questions. The proportion shown for each question is equal to the proportion of participants answering correctly (or correctly with the higher degree of certainty) to this specific question. Questions are ranked according to the number of correct responses at T0, from bottom to top. At T0, the proportion of correct answers ranged from 20 to 100%, with a proportion higher than 50% for most questions (90% of them) and was significantly lower than the level of chance (<0.5) for q5, q24, q25, and q26. The proportion of correct answers increased significantly between T0 and T1, except for q6, q8, q10, q13, q17, q23, and q30. For questions q10, q13, and q30, the proportion of correct answers was already close to 100% and thus could not increase. At T1, the probability of a correct answer was not significantly higher than 0.5 for q5, q17 or q26. The lowest proportion of correct answers at T1 was detected for q26 (allergens). The proportion of mastered answers significantly increased between T0 and T1 for all the questions.

### Parental Perception of the Content of the Brochure

The parents were on average very satisfied with the content of the brochure. The majority (98%) agreed that the content of the brochure was useful, easy to understand and answered their questions. Moreover, 98% of parents found that all the themes that they considered interesting were covered by the content of the brochure. Thirty-two percent of parents revealed that in the weeks prior to completing the questionnaire at T1 they looked for information on child feeding by means other than the brochure. Among those parents using other sources, the most popular ways to search information were via the internet (in particular websites on childcare, 37%), health care professionals (especially pediatricians, 28%) and parents' personal networks (grandparents, 19%, and friends, 16%). Regarding the three self-efficacy statements, a majority of parents (98%) declared they would try to follow the advice and recommendations contained in the brochure, but for 29% of them it would be difficult without the support of their partner and family. For 10% of parents, it would be difficult to follow the recommendations if their friends were not following the same ones. Whether parents would follow the recommendations did not differ according to parents' sociodemographic characteristics (age, parity, education level).

## Discussion

The main objective of this study was to evaluate the short-term effect of reading a brochure containing child feeding recommendations on the accuracy and the degree of certainty of French parents' knowledge. The results showed that knowledge accuracy, but especially knowledge certainty increased after parents read the brochure. At completion of the questionnaire, the parents with higher education levels were more certain of their correct knowledge compared to parents who had fewer years of formal education. For most of the questions, the proportion of correct answers significantly increased, as did the proportion of mastered answers for all questions. A secondary aim of the study was to evaluate parental attitudes toward the brochure. There was clear evidence that almost all parents were satisfied with the content of the brochure. Nevertheless, one-third of them sought child feeding information between T0 and T1 *via* means other than the brochure. The majority of parents were positive about following the recommendations of the brochure, but for one-third, it would be difficult to do so without the support of their partner and family. For some parents, following the advice of the brochure would be easier if their friends would also be willing to do so.

Our findings indicated that French parents already have a good level of knowledge regarding child feeding regardless of their age and parity; they scored high (median 22/30) even before having read the updated recommendations provided in the brochure. This may be seen as a result of the public health policy, first developed in 2001 regarding the National Program on Nutrition and Health (3). However, at T1, parents did not answer better than chance to q5 (all foods 4–6 m), q17 (family food) or q26 (allergens), meaning that sentences regarding those topics in the brochure might need reformulation or that parents might need more time or other consistent advice to gain more confidence with these recommendations. Parents with lower education levels, despite similar knowledge accuracy to parents with higher education levels, were less certain of their knowledge before reading the brochure. This interesting result could suggest that lack of confidence may interfere with parents' attitudes and actual knowledge and undermine the ability of parents with lower education to apply their knowledge regarding child feeding (35), thereby often displaying feeding practices less compliant with recommendations (15–18). In fact, higher education is often associated with better knowledge performance (36, 37). Knowledge accuracy significantly increased between T0 and T1 for all questions, except for q6 (new textures 6–8 m), q8 (food refusal), q17 (family food) and q23 (salt). For q26 (age of introduction of allergens), there was a significant increase, but the proportion of correct answers was still low after reading the brochure. For those questions, a reformulation of the related recommendations in the brochure might be advised to improve comprehension. Interestingly, the evolution in knowledge certainty was equivalent regardless of education level, which reveals that the design of the brochure was appropriate to reach its goal to inform all parents, regardless of their age, parity or education level, about best practices in child feeding.

This work introduces a novel approach to evaluate the effect of reading a brochure containing child feeding recommendations on the degree of knowledge certainty in parents. From the results, we highlighted an increase in both accuracy and certainty, but the increment of certainty was higher. Self-efficacy is defined as the belief in one's own capacity to perform a task or behavior (38), and it can transfer to different domains, including parents' ability to feed their child. According to Bandura's theory applied to the parenting domain, parents need to judge themselves efficacious in their parenting role to be successful and skillful in performing tasks related to that role (for example, feeding) (39). Also demonstrated in a study examining parental knowledge on child development is that the relationship between parental self-efficacy and proficiency in a given behavior is moderated by knowledge. In fact, when knowledge is high, self-efficacy and parenting competences (such as feeding) are positively associated (40). Parental self-efficacy and knowledge can both play a role in predicting parental behavior related to feeding, but this has not been extensively studied. Conrad et al. explored how accounting for both parental self-efficacy and knowledge could predict maternal behavioral competence (41). This study showed that, when there was high confidence, mothers who had more knowledge (vs. less) had more positive interactions with their children (41). Being certain in one's own knowledge might also strengthen parenting self-efficacy and contribute to the prediction of specific behaviors, but this aspect was not investigated in the present study, and further research is required to explore this point.

Evidence suggests that even if parents might have some basic nutritional knowledge and are aware of guidelines, they may still struggle to implement proper feeding practices due to factors, such as inconsistent and conflicting advice (42, 43). In fact, conflicting advice can create doubts as to whether your knowledge is the most up to-date or correct. A qualitative study conducted in Australia highlighted the perception of mothers that “everyone gives you advice” (43). Mothers can be influenced by their personal network in making decisions related to the introduction of solids, but they also do their own research, which may accentuate the perception of being surrounded by conflicting information (43). Parents' knowledge in relation to feeding is a fundamental basis on how to empower them to provide their children healthy foods and diets and favor healthy eating behavior. Strictly related to knowledge, there is the concept of health literacy, which is considered a health determinant and defined as “the degree to which individuals can obtain, process, and understand the basic health information and services they need to make appropriate health decisions” (44). While high health literacy levels might favor the adoption of healthier lifestyles (45), there is rising interest in a newer and more feeding-related concept, known as food literacy. Food literacy is defined as a set of food-related knowledge and skills that enables people to improve their own health by making informed choices about food and nutrition (46). This might be extended to parents making feeding choices for their children.

From a purely public health institutional perspective, the fact that the brochure was very welcomed by parents is an important achievement. The development of this kind of material requires a huge amount of time and the involvement of many different stakeholders (Minister of Health, research and public health institutions). In such processes, it is paramount to ensure proper vulgarization of messages that otherwise would not be fully understood from those that are the first recipients, in this case the parents. Despite the high satisfaction with the content of the brochure, 32% of parents declared they looked for information via other means. There might be different reasons for which parents still did so, but this was not explored in this study. First, since some topics were new, some recommendations may have surprised parents who needed to search for confirming information. Second, it is normal that some people need to double check information: a given piece of advice found on the internet may be more likely to be followed if it is also confirmed by a doctor. Third, not all the information needed by all parents can be present on a paper brochure, which has the purpose of giving information in a direct and synthetic way. For example, parents of premature children or children presenting with certain medical conditions might not find what they are looking for regarding their children's needs (30). Finally, some parents might still find it time-consuming to go and check one specific piece of information on the brochure, passing by all ages before finding what they needed. Parents might find it easier just to ask one specific question to a doctor or on an internet search engine, as examples. According to our present results, the most popular ways to search for information were via the internet (in particular websites on childcare), health care professionals (especially pediatricians) and parents' personal networks. This is in accordance with other studies (11, 30, 47) placing those three sources as the most used by parents when seeking child feeding information. It is important to simplify access to consistent information for parents from different and officially recognized sources.

Even if the majority of parents declared they would try to follow the advice and recommendations contained in the brochure, one-third of them thought it would be difficult to do so without the support of their partner and family. This aspect might limit the transition from knowledge to behavior. Parents' confidence in their role can also be defined as infant care self-efficacy, and it can impact the belief that parents can provide adequate and good care for their babies. Self-efficacy is also defined as one's judgment of how effectively one can deal with a designated situation. Self-efficacy influences people's thinking, feelings, motivations and actions (48). One of the sources to evaluate self-efficacy is verbal persuasion from others that for infant care translates into reinforcement from others (e.g., family, friends) (49). In fact, maintaining self-efficacy beliefs can be easier for those in whose capacities their significant others (e.g., family, friends) believe, thereby strengthening their belief to be doing well in the parent role (48). From our results, it can be deduced that parents may have low self-efficacy in relation to the verbal persuasion aspect. If encouragement from family and partner is lacking, this might exacerbate doubts that can lead to suboptimal care for the baby (49). More specific investigation is needed, using appropriate validated questionnaires to explore all four sources of information involved in the construction of parental self-efficacy [positive enactive mastery experiences, vicarious experiences, verbal persuasion and appropriate physiological and affective state (48)] regarding following recommendations.

This study is part of the brochure deployment process in order to validate the ability of the brochure to convey child feeding recommendation messages (before the national dissemination of the brochure). However, strengths and limitations must be considered alongside these results. First, the choice between different degrees of certainty that participants had to perform might have been impacted by the participants' individual capacity to estimate their own knowledge. In fact, estimating one's own knowledge is a task that people are not used to performing, and this kind of task often requires some training, which was not performed for our study. Additionally, one may raise the fact that knowledge is not always transformed into practice. Increasing knowledge may contribute to change behavior but further long-term studies are necessary to evaluate effective practices of parents. Even if, according to the knowledge-attitudes-behavior model, knowledge can impact attitudes and reflect on behavior, Eccles et al. tested multiple theoretical models trying to explain clinical behaviors, and, from their results, it appeared that knowledge was not predictive of behavior (21). However, in France, recent studies have shown that guidelines for feeding practices are generally followed in practice. In fact, a quantitative study including a sample of 600 parents showed that the majority adhered to recommendations on the introduction of solid foods (50), but other topics, such as milk feeding, were still not well integrated into parents' practices. Further studies will investigate the effect of the information contained in the brochure and whether the newly introduced recommendations will be integrated into parental feeding practices. The primary strength of our study lies in the novelty of its approach in the field of public health guideline evaluation. To the best of our knowledge, this is the first time that material intended for the general public has been evaluated for both knowledge accuracy and certainty before national dissemination. This study was included in a timely manner in the evaluation process of the brochure. The results allowed public health stakeholders to consider final adjustments about how information was given and organized in the brochure to be disseminated.

## Conclusions

Our results showed that, after reading a brochure containing the newly updated guidelines regarding child feeding, parents' knowledge increased. The knowledge increased both, in terms of accuracy and degree of certainty, despite a good level of knowledge at baseline, even for younger or less educated parents. The participants were generally satisfied with the content of the brochure, even if some of them expressed that they might experience some insecurity in following the recommendations without the support of their close family. Some parents felt the need to gather information from sources other than the brochure. From the perspective of programming a national plan for the dissemination of new child feeding recommendations it can be useful to provide parents with the same official information via differing sources (internet, health care professionals). This will contribute to avoiding rising doubts about how to perform optimal feeding practices and will make parents even more certain about their knowledge.

## Data Availability Statement

The raw data supporting the conclusions of this article will be made available by the authors, without undue reservation.

## Ethics Statement

The studies involving human participants were reviewed and approved by Institutional Review Board (IRB00003888, IORG0003254, and FWA00005831) of the French Institute of Medical Research and Health. The patients/participants provided their written informed consent to participate in this study.

## Author Contributions

SDR was a major contributor in writing the manuscript, the study design, conception of the questionnaires, and finalized the questionnaires. SN, PD, and CS contributed and validated the methodology and critically edited the questionnaires. SDR and CC analyzed the data. All authors critically reviewed and commented on subsequent drafts of the manuscript and approved the final version.

## Funding

The study was conducted as part of the project Edulia-Bringing down barriers to children's healthy eating, which has received funding from the European Union's Horizon 2020 research and innovation program under the Marie Skłodowska-Curie grant agreement No 764985. This work was also supported by grants from the Conseil Régional Bourgogne, Franche-Comte (PARI grant) and the FEDER (European Funding for Regional Economic Development).

## Conflict of Interest

The authors declare that the research was conducted in the absence of any commercial or financial relationships that could be construed as a potential conflict of interest.

## Publisher's Note

All claims expressed in this article are solely those of the authors and do not necessarily represent those of their affiliated organizations, or those of the publisher, the editors and the reviewers. Any product that may be evaluated in this article, or claim that may be made by its manufacturer, is not guaranteed or endorsed by the publisher.
