# Supplementary material for: Increasing Parental Knowledge About Child Feeding: Evaluation of the Effect of Public Health Policy Communication Media in France
Source: Front Public Health. 2022 Feb 24;10:782620. doi: 10.3389/fpubh.2022.782620 (PMC8907573; doi:10.3389/fpubh.2022.782620)
Supplement: Supplementary file 1 [file Data_Sheet_1.PDF]

Introduction ou 4e de couverture -

Nourrir son enfant, c'est bien sûr le faire manger, mais aussi guetter ses réactions, s'adapter, communiquer avec lui, l'accompagner vers l'autonomie... Les repas sont des moments forts dans la relation parents/enfant. Ce livret vous accompagne dans la **diversification alimentaire de votre enfant**, dès qu'il commence à prendre autre chose que du lait jusqu'à ce qu'il mange - presque - comme les grands.

Clé 1 - De la naissance à 3 ans, du lait, du lait, du lait !

Clé 2 - Entre 4 et 6 mois, commencez à donner de tout

Clé 3 - Etre attentif pour répondre au mieux aux besoins de son enfant

Clé 4 - A partir de 6/8 mois, des nouvelles textures à goûter, des nouveaux objets à manipuler

Clé 5 – Faire confiance à l'appétit de son enfant

Clé 6 - Privilégier le fait maison

Clé 7- A partir de 1 an, une alimentation équilibrée à la table familiale

Clé 8 - A partir de 2 ans, le refus de certains aliments

Clé 9 - Bouger, jouer et dormir

Clé 10 - En résumé : les groupes d'aliments à donner, des exemples de quantités, les aliments « interdits »

Clé 1 - De la naissance à 3 ans, du lait, du lait, du lait !

Jusqu'à l'anniversaire de ses 4 mois, un enfant ne doit prendre que du lait, sans rien d'autre. Entre 4 et 6 mois, en plus du lait, il va goûter d'autres aliments. Après 6 mois, son alimentation sera variée, mais toujours avec beaucoup de lait.

Allaiter, le moyen le plus naturel de débuter une alimentation santé

L'allaitement est une décision personnelle. Pour une mère, être soutenue par son entourage proche et avoir les conseils de professionnels formés facilite un bon démarrage. Il est recommandé d'allaiter jusqu'aux 6 mois de l'enfant mais même moins longtemps, c'est bénéfique à sa santé. Et si on veut prolonger, on peut donner le sein aussi longtemps qu'on le souhaite. Le tire-lait peut aider à continuer à allaiter à la reprise du travail.

Quand l'alimentation au sein est bien installée, on peut aussi alterner avec des biberons de préparation pour nourrisson (communément appelé « lait infantile ») en cas de besoin.

Quand on n'allaite pas ou plus

Donnez alors du « lait infantile » en poudre, 1er puis 2e âge, aux doses conseillées par le médecin traitant ou de PMI.

A partir de 1 an, donnez de préférence un « lait de croissance », qui est enrichi en fer (les formules en poudre sont moins chères). Vous pouvez aussi alterner avec du lait de vache entier UHT (ex : un biberon sur 2). Si vous donnez seulement du lait de vache entier UHT, il y a un risque que votre enfant manque de fer ; pour compenser il faudra alors lui donner des aliments riches en fer (légumes secs, viande) avec des aliments riches en vitamine C (agrumes) qui améliorent l'absorption du fer.

Les laits et produits laitiers ½ écrémés ou écrémés et 0 % MG ne sont pas adaptés aux nourrissons ; les laits et tous les produits à base de lait cru non plus.

Utilisez des « laits de croissance » et des produits laitiers nature, non aromatisés.

Le lait, et d'autres produits laitiers

Donner du yaourt et du fromage blanc non sucrés, un peu de fromage râpé de temps en temps à faire fondre dans les purées, c'est possible dès le début de la diversification, en plus de la tétée au sein ou du biberon. Il n'est pas nécessaire de donner des produits laitiers « spécial bébé » qui sont plus chers.

Les faux « laits »

Les « laits d'amande » et autres boissons végétales, même enrichis en calcium, ne sont pas du lait et ne répondent pas aux besoins nutritionnels d'un jeune enfant. Le « lait » de soja et les autres produits à base de soja sont déconseillés avant 3 ans.

Clé 2 - Entre 4 et 6 mois, commencez à donner de tout

Légumes, fruits, volaille, poisson, viande, œufs, légumes secs (lentilles, haricots, pois chiches…), féculents (pâtes, riz, semoule, pain), produits laitiers, fruits à coque non salés, matières grasses...
Un bébé peut commencer à découvrir toutes les familles d'aliments entre 4 et 6 mois. On sait aujourd'hui que c'est la meilleure période pour que son organisme accepte les aliments en cause dans les allergies. Vous pouvez cependant demander l'avis de votre médecin traitant ou de PMI s'il y a des allergies dans la famille.

Le saviez-vous ?

N'hésitez pas à proposer des légumes secs (lentilles, pois chiches, haricots secs) en purée lisse.

Les fruits à coque non salés (amandes, noix, noisettes) sont riches en oméga 3. Achetés en poudre, une cuillère à café peut être mélangée de temps en temps dans une purée.

Le bon moment ? C'est le vôtre et celui de votre bébé
Commencez quand vous le souhaitez, mais en tout cas pas après 6 mois : dès l'anniversaire des 4 mois ou un peu plus tard, en particulier si votre enfant est prématuré. Si c'est le cas de votre enfant, demandez un avis médical.
Vous pouvez commencer au repas qui convient le mieux à votre organisation familiale. Si votre enfant est gardé à l'extérieur, cela peut tout à fait être le soir.

A la cuillère, les purées et les compotes bien lisses
On commence en général à donner à manger à la cuillère. Proposez tous les aliments en purée ou en compote lisses pour que le bébé puisse les avaler sans risque. Les soupes moulinées peuvent aussi être proposées à la tasse.
Si votre enfant veut découvrir les aliments avec les doigts, laissez-le faire, même si c'est un peu salissant… Il les aimera d'autant plus !

Faire découvrir un goût à la fois
Au début, il vaut mieux donner chaque nouvel aliment séparément. Par exemple, quelques cuillères à café de purée d'un légume, de purée de volaille, de compote… Votre enfant acceptera encore mieux les nouveaux aliments si vous variez les légumes, les fruits, etc. au fil de la semaine, pour lui faire découvrir différents goûts et couleurs.

Une fois que chaque aliment a été accepté seul, vous pouvez proposer les aliments mélangés (purée de légumes et pomme de terre, purée de légumes et volaille, etc.)

Donner des matières grasses
Mélangez 1 cuillère à café de matière grasse dans l'équivalent d'un petit pot au repas dès le début de la diversification.

Petit encadré : **Préparer une purée lisse**

Pour les légumes, les légumes secs, les pommes de terre : cuire à l'eau ou à la vapeur, sans sel. Pour la volaille, la viande ou le poisson : bien cuire à cœur, sans sel.

Mixer les aliments ou les écraser très finement à la fourchette. Ajouter un peu d'eau fraîche et de la matière grasse (huile ou beurre). Mélanger pour obtenir une consistance bien lisse. Ne pas saler.

Astuce : congeler une partie de la purée dans un bac à glaçons recouvert de film alimentaire, pour utiliser au fur et à mesure des repas. Placez-le dans une poche plastique datée et identifiée.

Sinon, on peut conserver la purée au maximum 24 h au réfrigérateur dans une boîte hermétique en verre.

Si on utilise un petit pot du commerce, on vérifie sur l'étiquette la durée de consommation après ouverture.

Les quantités : proposer sans forcer
Au début, votre bébé ne prend que quelques cuillérées car son estomac est petit, suivies de la tétée à volonté ou du biberon dans les quantités indiquées par votre médecin. Peu à peu, il va manger plus : fiez-vous à son appétit, observez ses réactions, ne le forcez jamais. Il n'y a pas de quantité à atteindre, chaque enfant est différent.

S'il refuse un nouvel aliment, n'insistez pas mais réessayez quelques jours plus tard. Il faut souvent présenter plusieurs fois le même aliment (parfois jusqu'à 10 fois !) pour qu'un enfant l'accepte et commence à y prendre plaisir. Tenez compte de son humeur… et de la vôtre !

Si votre enfant a du mal les premiers jours à accepter autre chose que son lait, ce n'est pas grave, tant qu'il boit du lait ! Mais rappelez-vous qu'après 6 mois, le lait seul ne suffit plus à couvrir ses besoins.

Pour se repérer et à titre indicatif
Consultez le tableau d'introduction par groupes d'aliments page 00.

La bonne position
Pour éviter une fausse route (le fait d'« avaler de travers »), présentez la cuillère par en dessous, c'est-à-dire en la portant du menton de l'enfant vers sa bouche.

Pour les fruits
Cuire un fruit de saison épluché à la casserole avec 2 cuillères d'eau puis mixer, sans ajouter de sucre ; Puis, progressivement, proposez des fruits tendres crus ou cuits écrasés à la fourchette, toujours sans sucrer.

L'alimentation végétalienne ou vegan (sans aliments d'origine animale) est dangereuse pour les jeunes enfants, car elle peut entraîner des carences importantes en fer, protéines, calcium, vitamines...

Clé 3 – Etre attentif pour répondre au mieux aux besoins de son enfant

Accompagner l'évolution de votre enfant
Chaque nouveauté est une étape ! Un aliment inconnu, des morceaux à mâcher, une cuillère à attraper… autant de situations qui vont entraîner des réactions de votre enfant. Pour bien s'adapter à ses besoins, le mieux est de l'observer. A-t-il l'air surpris ? Content ? Votre rôle de parent est de le laisser expérimenter des aliments, à son rythme, en lui montrant qu'il peut avoir confiance.

L'encourager et être patient
Si votre enfant est surpris par l'odeur, la couleur, le goût ou la texture d'un aliment, il faut le rassurer en lui parlant de cet aliment. Une petite grimace ne doit pas vous empêcher de refaire un essai. Ne le forcez pas, mais proposez-lui quelques jours plus tard ce qu'il a refusé : petit à petit, il se familiarisera avec la nouveauté, la goûtera... et finira sans doute par l'apprécier. Moins vous vous braquerez, plus il y a de chances que votre enfant goûte avec plaisir l'aliment quelque temps plus tard.

Attention cependant à distinguer le refus d'un aliment nouveau avec un véritable dégoût : comme vous, votre enfant peut ne pas aimer certains produits. Il ne faut jamais le forcer à manger.

Bannir les écrans pendant le repas
Pour que le repas soit un moment d'échange, on éteint télé, tablette et on laisse son téléphone à distance. A l'heure du repas comme à beaucoup d'autres moments, votre enfant a besoin de toute votre attention. En étant pleinement présent, vous captez tous les signaux qu'il envoie et vous pouvez y répondre au mieux.

Clé 4 - A partir de 6/8 mois, des nouvelles textures à goûter, des nouveaux objets à manipuler

Faire découvrir de nouvelles textures…
A partir de 6/8 mois (et pas après 10 mois), selon l'âge auquel vous avez commencé à diversifier, c'est important de proposer progressivement de nouvelles textures à votre enfant. Il va ainsi pouvoir développer ses capacités à bien mâcher.
Introduisez une seule nouvelle texture lors d'un repas. Si vous donnez des petits pots, variez les marques et les recettes, la consistance et la taille des morceaux varient d'un produit à l'autre.

… et expérimenter de nouveaux « ustensiles »
Votre enfant va tester la cuillère, la tasse… plus tard ce sera la fourchette... Laissez-le aussi prendre en main des morceaux tendres afin qu'il se familiarise avec : il va les malaxer, les suçoter, les mâchouiller puis les avaler.

Vous pouvez aussi lui donner de l'eau au verre, en l'aidant pour boire.

Il est important que votre enfant soit bien assis et sous votre regard pendant toute la durée du repas

Tous à table !
Même s'il ne mange pas comme le reste de la famille, vous pouvez dès cet âge installer votre bébé à table avec vous pour partager ce temps convivial.

Quelques signes qu'un enfant est prêt pour de nouvelles textures

- Il avale les purées lisses et épaisses sans problème
- Il maintient sa tête et son dos droits dans sa chaise
- Il fait des mouvements de mâchonnement quand il porte quelque chose à sa bouche
- Il est capable de tenir un aliment et de le porter à sa bouche (il cherche à se nourrir seul)
- Il essaye de prendre des aliments dans votre assiette

A la crèche ou chez la nounou
N'hésitez pas à parler des habitudes alimentaires que vous souhaitez donner à votre enfant avec les personnes qui le gardent. C'est important qu'on respecte vos choix.

Ordre des différentes textures à proposer progressivement de 6/8 mois jusqu'à 3 ans

1. Tous les aliments hachés ou écrasés grossièrement à la fourchette (purée granuleuse)
2. Purées dans lesquelles on ajoute des pâtes alphabet, de la semoule, du riz bien cuit, de petits morceaux de volaille, de viande tendre ou de poisson très cuits, de l'œuf dur haché
3. Aliments en morceaux très mous qui s'écrasent entre la langue et le palais ou entre les doigts (ex. banane)

4. Aliments en morceaux à croquer, à mâcher avec les dents (aliments cuits, fruits crus, crudités en petits morceaux…)

Le saviez-vous ?
Même sans dents, un enfant peut mastiquer des morceaux mous, fondants.

Pour vérifier la texture d'un aliment, vous pouvez tester vous-même la texture et voir s'il s'écrase contre le palais avec la langue ou entre les doigts

Ne donnez pas d'aliments petits, durs et ronds (type cacahuète) à un enfant de moins de 5 ans, il risquerait de s'étouffer.

Coupez toujours les aliments mous et ronds ou ovales en deux avant de les donner.

Astuce : Proposer un quignon de pain de temps en temps au repas permet de stimuler la mastication et de fortifier la mâchoire. Au début il est préférable de remplacer le morceau de pain lorsqu'il est trop imbibé de salive car un morceau risque de se détacher.

Clé 5 - Faire confiance à l'appétit de son enfant

Quoi et quand
Avec le temps, un enfant mange plus aux repas mais fait moins de repas par jour. Avec la diversification, un enfant, petit à petit ou du jour au lendemain, se met spontanément à manger cinq puis quatre fois par jour. A partir d'environ 8 mois, il va prendre un petit déjeuner, un déjeuner, un goûter et un dîner.

Pour des conseils indicatifs, allez à la **Clé 10**.

A partir de 1 an, en plus du lait, un enfant mangera chaque jour des aliments de chaque grand groupe d'aliments : légumes, fruits, viande ou poisson (ou légumes secs deux fois par semaine), féculents, produits laitiers. Par exemple, volaille, poisson ou viande et légumes avec un peu de matière grasse à midi et produit laitier et féculents et fruits au goûter. Ou, aussi bien, volaille ou poisson, légumes et féculents avec un peu de matière grasse à midi et produit laitier et fruit au goûter.

Vous savez quoi lui donner et quand, mais votre enfant sait combien
Dès sa naissance, un bébé prend naturellement les quantités de lait dont son corps a besoin. Et cela continue après quand vous lui proposez d'autres aliments. Au début, il goûtera quelques cuillérées à café, puis progressivement, son appétit et ses capacités de digestion augmentent. Augmentez les quantités que vous lui proposez, en vous aidant des moyennes proposées dans les menus p. 00 et en respectant son appétit.
Il est possible qu'au moment où il commence à manger tout seul, votre enfant mange moins. N'insistez pas : il sait quand il n'a plus faim.
Evitez de lui donner à manger en dehors des repas « pour compenser » ; en ne mangeant qu'aux repas, il lui sera plus facile d'être lui-même à l'écoute de son appétit.

Mais si vous trouvez que votre enfant mange vraiment trop ou pas assez, parlez-en au médecin traitant ou de PMI.

Quelques signes de faim du nourrisson :
Il pleure, agite vivement bras et jambes, ouvre la bouche quand le biberon ou la cuillère approche.
Quelques signaux de rassasiement :
Il s'endort sur le sein ou le biberon ou ralentit sa tétée ou sa prise de nourriture, tourne la tête quand on lui tend la cuillère, regarde ailleurs

Il pleure sans arrêt
Est-ce vraiment à manger qu'il demande ?
Ou veut-t-il mâchouiller pour calmer ses dents qui sortent ? Ou attirer notre attention ?

Un repère : la courbe de croissance du carnet de santé
Comme les adultes, certains enfants mangent plus - ou moins - que d'autres. Les consultations systématiques de suivi avec le médecin traitant ou à la PMI servent à vérifier que le poids et la taille de votre enfant suivent une évolution normale. Au moindre doute, vous pouvez aussi consulter entre deux consultations systématiques.

Pour vous donner une idée, exemples de menus, voir Clé 10 (PARTIE MANQUANTE).

Clé 6 – Privilégier le fait maison

Pour une plus grande variété de goûts et de textures
Cuisiner maison permet d'utiliser des produits de saison, locaux et des produits de sa culture culinaire. Cela permet aussi de doser les quantités de matières grasses ou de sucre par exemple. Si possible, on privilégie le bio pour les fruits et légumes, les féculents complets (riz, pâtes, semoule complets…) et les légumes secs. On lave les fruits et légumes soigneusement et, s'ils ne sont pas bio, on les épluche.

Des conseils et astuces sur mangerbouger.fr et sur la brochure 50 petites astuces pour manger mieux et bouger plus

Les aliments surgelés peuvent être pratiques, mais prenez alors les produits nature, non cuisinés ; par exemple des légumes épluchés et en morceaux, des fruits, des filets de poisson nature….

Jusqu'à 3 ans, on évite les plats industriels préparés pour adultes et les produits ultra-transformés qui sont souvent gras, sucrés ou salés, sans parler des additifs (colorants, conservateurs…) dont on ne connaît pas l'impact éventuel sur la santé.

Bon à savoir
Assaisonnez avec des aromates et des herbes pour leur donner du goût et éveiller l'appétit.

Les petits pots et les plats préparés du commerce pour bébés
On peut en donner de temps en temps quand on n'a pas le temps de cuisiner, hors domicile, en voyage... Si le petit pot ne contient pas de matière grasse, on y ajoute une cuillère à café d'huile de colza, de noix ou d'olive ou une noisette de beurre. Les aliments du commerce pour les moins de 3 ans sont strictement contrôlés sur le plan de l'hygiène et de la fabrication.

Bon à savoir
Ne pas saler ni sucrer ce que vous donnez à votre enfant jusqu'à 3 ans,
que ce soit des aliments « faits maison » ou du commerce.

La seule boisson indispensable : l'eau
L'eau du robinet convient aux bébés et aux jeunes enfants (sauf exception signalée par la mairie). Si vous utilisez de l'eau en bouteille, vérifiez sur l'étiquette qu'elle convient aux nourrissons.

Proposez de l'eau nature, à la tasse ou au verre.

Les jus de fruits quels qu'ils soient sont trop sucrés ; ils ne sont pas recommandés. Ne pas non plus donner de sodas (même light).

Bien veiller à l'hygiène
- Nettoyage et entretien régulier des surfaces de travail, des matériels et des ustensiles.
- Nettoyage du frigo à chaque fois que des aliments ont souillé des surfaces.
- Maintien de la température à 4°C maximum dans la zone la plus froide du réfrigérateur (avec un thermomètre de frigo)

*Pour plus de précisions sur l'hygiène alimentaire et en cuisine, rendez-vous sur le site **agirpourbébé.fr***

Clé 7 - A partir de 1 an, une alimentation équilibrée à la table familiale

Prendre ou reprendre de meilleures habitudes pour toute la famille
Peu à peu, votre enfant va manger presque la même chose que toute la famille, en quantités adaptées à son âge. D'où l'importance que votre alimentation soit variée et équilibrée. Vous l'avez expérimenté, la grossesse et l'arrivée d'un enfant sont souvent l'occasion d'adopter ou de reprendre des habitudes plus saines… à garder au long cours.

*Retrouvez les recommandations pour l'alimentation des enfants et des adultes, des recettes et des idées de menus sur **mangerbouger.fr**.*

Toujours beaucoup de lait
Jusqu'à 3 ans, continuer à donner à votre enfant beaucoup de lait ; l'allaitement maternel est toujours possible; sinon donnez environ ½ litre par jour de « lait de croissance » de préférence, ou de « lait de croissance » alterné avec du lait de vache entier UHT ; à défaut donnez du lait de vache entier UHT seul (voir Clé 1).

3 repas par jour + 1 goûter (et 1 seul)
Dorénavant et pendant toute l'enfance, donnez 3 repas par jour et un goûter. Au goûter, proposez une tranche de pain et un fruit ou une compote ou un produit laitier ; évitez les viennoiseries ou autres aliments sucrés et gras.

- Ne redonnez pas un goûter si l'enfant en a déjà eu un à la crèche ou chez la nounou !
- Aux repas, servir de petites quantités et ne pas resservir sauf si votre enfant le demande.
- En dehors des repas, ne laissez pas de nourriture visible et encore moins à portée de main.
- Ne laissez pas votre jeune enfant se servir seul dans le placard ou le réfrigérateur.

Les biscuits pour bébé et autres aliments sucrés proposés dans le commerce pour les moins de 3 ans sont trop gras et sucrés.

Clé 8 - A partir d'environ 2 ans : le refus de certains aliments

Il ne veut pas ou plus de certains aliments, surtout les légumes…
Jusqu'à environ 2 ans, la plupart des enfants acceptent de nouveaux aliments même s'il faut parfois plusieurs essais pour qu'ils s'y habituent. Ensuite, et parfois pendant plusieurs années, beaucoup d'enfants deviennent plus « difficiles ». 2 ans, c'est aussi l'âge où la plupart commencent à affirmer leur autonomie, à dire non à tout ; un enfant peut même rejeter des aliments qu'il appréciait avant, en particulier les légumes.

Être patient…
Pas de panique, cette opposition est normale. Le mieux est de s'armer de patience : ça va passer ! Inutile de forcer votre enfant à manger ou à finir son assiette. Le repas doit rester un moment de plaisir, pas de combat. Votre enfant aura l'occasion de goûter à nouveau l'aliment la prochaine fois qu'il sera au menu familial, peut-être sous une autre forme.

… mais poser des limites
C'est vous qui décidez de la composition du repas, pas votre enfant. S'il refuse de goûter un plat, évitez de compenser en donnant plus de dessert ou un gros goûter. Parlez-en aussi avec les personnes qui le gardent.

Comme vous, un enfant a ses préférences
La variété de l'alimentation favorise une bonne santé ; mais si vous voyez que votre enfant aime beaucoup quelque chose, rien n'empêche de le mettre au menu plusieurs fois dans la semaine.

Eviter le « chantage affectif »
Bannir les formules comme « *Encore une petite cuillère pour me faire plaisir* » ou « *Si tu finis ton assiette, tu auras un bon dessert* ». Un enfant ne doit pas manger pour faire plaisir à ses parents ou pour avoir une récompense, mais parce qu'il a faim.

Manger plus de légumes en famille
Manger est un plaisir contagieux ! Les enfants reproduisent les comportements qu'ils observent. En mettant plus de légumes au menu de toute la famille, frères et sœurs compris, on montre (et on dit) à notre enfant qu'on aime ça. Une façon de motiver toute la famille !

Jouer sur la diversité
- Un même légume n'a pas le même goût ni le même aspect en salade, en tarte ou dans un pot-au-feu.
- Mettez de la couleur en ajoutant des légumes (courgettes, brocolis, tomates, etc.) ou un peu de sauce tomate dans les pâtes… Attention, le ketchup n'est pas un légume, mais une sauce qui contient du sucre !

Découvrir ensemble les fruits et légumes
- Aller au marché ou dans une grande surface avec son enfant peut aussi être l'occasion de lui faire découvrir les différentes formes et couleurs des fruits et légumes. On nomme les produits, on lui fait toucher le velouté des pêches…
- Si on n'a pas de jardin, faire pousser du basilic ou des tomates en pot sur le balcon permet à un enfant de se familiariser avec des odeurs et des formes

Clé 9 – Bouger, jouer et dormir

Votre enfant évolue sans cesse…

Au fil des mois, un enfant progresse de façon impressionnante : il sait maintenir sa tête et son dos droit, se tenir debout avec un appui, se déplacer à quatre pattes, avant de marcher puis de courir… Il aime bouger et en a besoin, alors laissez-le faire ou sollicitez-le, notamment par des jeux, plusieurs fois par jour.

Dès le plus jeune âge, bouger

A partir du moment où l'enfant peut tenir sa tête, il est conseillé de le mettre au moins 30 minutes à plat ventre plusieurs fois par jour pendant qu'il est éveillé.

A partir de 6 mois

- Allongez votre enfant sur le dos sur un revêtement matelassé (tapis, moquette, etc.). Posez à côté de lui, dans son champ de vision, un objet très coloré, brillant, ou qui fait de la musique. Attiré par cet objet, votre enfant va se retourner et ramper pour l'attraper.
- Tenez votre enfant debout et laissez-le prendre appui fermement sur ses jambes.
- À la maison, laissez-le se mouvoir et découvrir les objets autour de lui en étant présent et attentif, en l'encourageant et en vérifiant bien tout pour sécuriser son environnement : prises, portes, petits objets, produits dangereux…

À partir de 12 mois

Créez un parcours avec des cerceaux, des petites marches, etc. Aidez-le à suivre les différentes étapes.

A partir de 2 ans

- Votre enfant découvre son corps et commence à jouer avec les autres. L'emmener régulièrement au parc ou dans un square qui offre des structures adaptées à son âge lui permettra de rencontrer d'autres enfants pour jouer.
- Jouez au ballon en famille : asseyez-vous en rond et faites rouler le ballon vers les autres joueurs, sur le sol.
- Faites découvrir à votre enfant les joies du tricycle, de la trottinette ou encore des patins à roulettes pour qu'il découvre de nouvelles sensations, améliore son endurance, son équilibre et son orientation dans l'espace.
- Emmenez-le à la piscine pour qu'il se familiarise avec l'eau à son rythme.

Avant 5 ans, il est recommandé qu'un enfant « bouge » au moins 3 heures par jour, par des activités variées et ludiques, et aussi souvent que possible en plein air : marcher, courir, sauter, lancer une balle, voire nager…

*Pour toutes les recommandations concernant l'activité physique des jeunes enfants, consultez les sites **mangerbouger.fr** et **agirpourbébé.fr.***

Pas d'écrans pour les jeunes enfants...

Evitez de mettre votre enfant devant un écran (télévision, tablette, smartphone) avant l'âge de 3 ans, ni dans une pièce où la télévision est allumée, même s'il ne la regarde pas. C'est une période très importante d'apprentissage, notamment du langage, et les mauvais effets des écrans sur le langage, le sommeil, la corpulence ne se voient pas immédiatement mais apparaîtront plus tardivement. Jouer « en vrai » avec son enfant est la meilleure façon de favoriser son développement.

Le sommeil

De la naissance à 1 an, il est recommandé qu'un enfant dorme entre 12 et 16 heures par 24 h ; puis, jusqu'à l'âge de 5 ans, entre 11 et 14 h par 24 h. Il est important de respecter une régularité dans les rythmes de sommeil (heures de coucher, de sieste…).

- Ne pas associer endormissement et alimentation : par ex. ne pas laisser l'enfant s'endormir dans son lit avec un biberon.
- Ne pas associer endormissement et écran : pas d'écran allumé dans la pièce où dort l'enfant.

Clé 10 - En résumé : les groupes d'aliments à donner, les quantités moyennes, les aliments « interdits »

- **TABLEAU RECAPITULATIF D'INTRODUCTION DES ALIMENTS ET TEXTURES A VENIR : Voir le tableau à la page suivante**

Aliments non adaptés aux enfants de moins de 3 ans

- Pas de miel pour les enfants de moins d'un an
- Pas de café, de thé, de sodas caféinés ni de boissons dites « énergisantes » car contiennent de la caféine
- pas de produits et boissons contenant des édulcorants ou faux sucres (les produits « light »)
- pas de viandes crues ou peu cuites
- pas de lait cru ou de fromages au lait cru, à l'exception des fromages à pâte pressée cuite comme le gruyère ou le comté
- pas d'œufs crus ni produits à base d'œufs crus ou peu cuits (tels que les mousses au chocolat et mayonnaises faites maison) ;
- pas de coquillages ni poissons crus.

Aliments déconseillés

- Chocolat et produits chocolatés (contiennent trop de nickel pour eux)
- limiter les produits à base de soja

Supplémentations alimentaires

En dehors d'une prescription de votre médecin (en vitamine D, K par exemple), ne donnez pas de vous-même de supplémentation en vitamines ou minéraux à votre enfant.

De la naissance à l'anniversaire des 4 mois :

Donner exclusivement du lait, au sein ou en préparation pour nourrissons (« lait infantile »)

**Groupes d'aliments**	**Conseils généraux adaptés à toutes les tranches d'âge**	**Entre 4 et 6 mois**	**A partir de 6/8 mois**	**De 1 à 3 ans**
		Débuter la diversification<br>Proposer au cours de cette période tous les groupes d'aliments<br>Sous forme mixée lisse<br>En cas d'allergies dans la famille, consulter le médecin avant de débuter.	Faire découvrir progressivement toutes les textures pour développer les capacités de l'enfant à mâcher : des purées lisses aux aliments hachés, écrasés, aux petits morceaux mous	Donner une alimentation variée et équilibrée proche de celle recommandée pour les adultes, en quantités adaptées<br>Donner toujours du lait
**Lait maternel<br>Et/ou<br>Préparations infantiles**		Lait maternel<br>ou<br>Transition « lait infantile » 1er âge au « lait infantile » 2e âge	Lait maternel<br>et / ou<br>« Lait infantile » 2e âge : 500 ml/j	Lait maternel<br>et / ou<br>« Lait de croissance » : 500 ml/j ou équivalents en produits laitiers<br>et/ou lait de vache entier : 500 ml/j ou équivalents en produits laitiers
**Produits laitiers<br>(fromage blanc, yaourts, fromage *en alternance*)**	- Nature, non aromatisés, non sucrés<br>- Privilégier les fromages les moins salés<br>- Ne pas donner de lait et fromages au lait cru (possibles : fromages à pâte pressée cuite comme le gruyère ou le comté)<br>- Les produits « 0% » ou light ne conviennent pas aux bébés<br>- Les produits « spécial bébé » n'ont pas d'intérêt particulier	Par exemple :<br>- quelques cuillères à café de yaourt au goûter<br>- de temps en temps, faire fondre un peu de fromage râpé dans une purée	A partir de 8/10 mois, petits morceaux de fromages mous<br>A partir de 10 mois morceaux de plus en plus durs	Toutes les textures<br>Un yaourt équivaut à 20 g de fromage ou à 150 à 200 ml de lait<br>Ne pas dépasser 800 ml de lait/j ou son équivalent
**Légumes**<br>*Tous*	- Plutôt de saison<br>- Plutôt produits localement<br>- Bio si possible	A proposer tous les jours<br>Donner quelques cuillères à café jusqu'à l'équivalent d'1 petit pot selon l'appétit de l'enfant<br>Bien cuits	A proposer à chaque repas et donner selon l'appétit de l'enfant<br>Bien cuits	A proposer à chaque repas et donner selon l'appétit de l'enfant<br>Crus et cuits, toutes textures, dont morceaux à croquer
**Fruits**<br>*Tous*	- Plutôt de saison<br>- Plutôt produits localement<br>- Bio si possible	A proposer tous les jours et donner selon l'appétit de l'enfant<br>Bien cuits et mixés en compotes, non sucrées	A proposer à chaque repas et selon l'appétit de l'enfant<br>Fruits bien mûrs ou fruits cuits non sucrés	Crus et cuits, toutes textures, dont morceaux à croquer<br>Doivent être donnés à chaque repas<br>Selon l'appétit de l'enfant
**Fruits à coque : noix, noisettes, amandes,** *non salés*	En poudre uniquement, pour éviter les risques d'étouffement<br>Non salés uniquement	Mélangés à d'autres aliments<br>1 cuillère à café en poudre		
**Légumes secs : lentilles, haricots secs, pois chiches, etc.**	Bio si possible<br>Adapter en fonction de la façon dont l'enfant les digère	A proposer de temps en temps<br>1 cuillère à café<br>Bien cuits et mixés en purée lisse	Proposer environ 1 fois par semaine<br>Quelques cuillères à café<br>Progressivement,<br>purée lisse puis purée écrasée à la fourchette<br>puis légumes secs entiers toujours bien cuits	Donner des légumes secs au moins 2 fois par semaine<br>Toutes les textures
**Féculents : Pommes de terre, pâtes, riz, semoule, pain,**	Bio si possible<br>Adapter en fonction de la façon dont l'enfant les	Le gluten peut être introduit dès 4/6 mois même pour les enfants à risque	1/2 féculents / 1/2 légumes	A proposer tous les jours

**et féculents complets (riz complet ou semi complet, pâtes complètes ou semi complètes, etc.)**	digère pour les produits complets	d'allergie<br><br>¼ féculents mixés / ¾ légumes mixés<br><br>Toujours associés avec des légumes et des matières grasses	Toujours associés avec des légumes et des matières grasses<br>Riz, semoule, pâtes de moins en moins fines, pain<br>pommes de terre en petits morceaux fondants	Toutes textures<br><br>3 à 4 cuillères à soupe par jour
**Volaille, poisson, autres viandes et œufs**	Privilégier la volaille et le poisson (poisson 2 fois par semaine dont 1 poisson gras : sardine, maquereau, saumon…)<br><br>Toujours très cuits	Pas forcément tous les jours<br><br>1 cuillère à café, aliments mixés, dans la purée de légumes et/ou féculents	10 g/jour = 2 cuillères à café<br>ou<br>¼ d'œuf dur	Hachés, écrasés, puis progressivement en morceaux<br>Entre 1 et 2 ans : 20 g/j = 4 cuillères à café ou 1/3 d'œuf dur<br>Entre 2 et 3 ans : 30 g/j = 6 cuillères à café ou ½ œuf dur
**Matières grasses : huile (colza, noix, olive), beurre**	A ajouter crues aux préparations maison ou aux petits pots du commerce, en privilégiant l'huile	1 cuillère à café mélangée à un repas diversifié (1 fois par jour)	1 cuillère à café d'huile ou 1 noisette de beurre à chaque repas diversifié<br>(2 fois par jour)	2 cuillères à café d'huile ou 1 noisette de beurre par jour
**Boissons : eau et boissons sucrées (dont tous les types de jus de fruits)**	La seule boisson recommandée est l'eau nature	A volonté, au verre<br>L'eau du robinet convient aux jeunes enfants, sauf avis contraire de la mairie		
		Eviter les boissons sucrées, dont tous les jus de fruits<br><br>Eviter d'ajouter du sirop dans l'eau<br><br>Ne pas donner de boissons « light »		
**Charcuterie**		Jambon blanc possible de temps en temps, mixé :<br><br>1 cuillère à café	Jambon blanc possible de temps en temps, mixé puis en petits morceaux :<br><br>10 g = 2 cuillères à café	Jambon blanc possible de temps en temps :<br><br>Entre 1 et 2 ans : 20 g/j = 4 cuillères à café<br><br>Entre 2 et 3 ans : 30 g/j = 6 cuillères à café
**Sel et produits salés**		Ne pas saler le fait maison ni les petits pots du commerce<br>Ne pas donner de produits salés (produits apéritifs…)		Ne pas saler les produits du commerce<br>Limiter la consommation de produits salés (produits apéritifs…)
**Produits sucrés : confiseries, gâteau, crèmes dessert, glaces, ketchup, pâte à tartiner…**		Surtout pas de miel avant 1 an (risque microbiologique)<br>On peut sucrer les produits laitiers nature avec des fruits<br>Les aliments sucrés du commerce ciblant les moins de 3 ans et les biscuits pour bébé n'ont pas d'intérêt nutritionnel		A limiter<br>Limiter les produits chocolatés du fait de la teneur en nickel du chocolat<br>Les céréales du petit-déjeuner sont en général sucrées, voire sucrées et grasses

Produits à éviter/limiter/ne pas donner
